# Temporal bisection is influenced by ensemble statistics of the stimulus set

**DOI:** 10.3758/s13414-020-02202-z

**Published:** 2020-11-26

**Authors:** Xiuna Zhu, Cemre Baykan, Hermann J. Müller, Zhuanghua Shi

**Affiliations:** General and Experimental Psychology, Department of Psychology, LMU Munich, 80802 Munich, Germany

**Keywords:** Temporal bisection, Stimulus spacing, Central tendency, Ensemble perception

## Abstract

Although humans are well capable of precise time measurement, their duration judgments are nevertheless susceptible to temporal context. Previous research on temporal bisection has shown that duration comparisons are influenced by both stimulus spacing and ensemble statistics. However, theories proposed to account for bisection performance lack a plausible justification of how the effects of stimulus spacing and ensemble statistics are actually combined in temporal judgments. To explain the various contextual effects in temporal bisection, we develop a unified *ensemble-distribution account* (EDA), which assumes that the mean and variance of the duration set serve as a reference, rather than the short and long standards, in duration comparison. To validate this account, we conducted three experiments that varied the stimulus spacing (Experiment 1), the frequency of the probed durations (Experiment 2), and the variability of the probed durations (Experiment 3). The results revealed significant shifts of the bisection point in Experiments 1 and 2, and a change of the sensitivity of temporal judgments in Experiment 3—which were all well predicted by EDA. In fact, comparison of EDA to the extant prior accounts showed that using ensemble statistics can parsimoniously explain various stimulus set-related factors (e.g., spacing, frequency, variance) that influence temporal judgments.

We as humans have the ability to perceive the passage of time relatively accurately. Our sense of time allows us to adapt to and interact with a dynamic external world. As has been suggested in classical ‘internal-clock’ models (Gibbon, Church, & Meck, 1984; Treisman, 1963), our ability to time (experienced) events in the external world is based on an internal timer. Although there is no physical timer in our brain, behavioral studies have shown the internal-clock model can explain many empirical findings and predict the key feature of time perception: the scalar property (i.e., the Weber scaling). However, more and more evidence shows that, even though we are well capable of time measurement, we are still prone to biases in our timing that depend on both internal states (e.g., mental load, attention, emotional state) and external contexts (Allman & Meck, 2012; Allman, Teki, Griffiths, & Meck, 2014; Pronin, 2013).

One prominent contextual bias in time perception, which has been puzzling for more than a century and half, is the central-tendency effect: Duration judgments are assimilated to the center of the sample durations. Thus, for example, when asked to reproduce a series of time intervals, participants judge *long* durations as being shorter and *short* durations as being longer than they actually are. This was first, and accidentally, discovered by Karl von Vierordt (Lejeune & Wearden, 2009; von Vierordt, 1868), who misused the “method of average error” that Fechner had devised by implementing randomization instead of repeated measures (Glasauer & Shi, 2018). The central-tendency effect is one classical example showing that the ensemble mean derived from long-term memory of sampled stimuli strongly influences perceptual judgments. Recent studies have suggested that ensemble statistics can be rapidly computed from a set of variant objects or a sequence of events (Alvarez, 2011; Ariely, 2001; Chen, Zhou, Müller, & Shi, 2018; Whitney & Yamanashi Leib, 2018). For example, we can quickly estimate the average size of apples in a basket, or the average tempo of a piece of music. It has been suggested that the use of ensemble statistics is beneficial by enhancing the reliability of sensory estimates (Alvarez, 2011). With regard to the central-tendency bias, this has been confirmed by Bayesian modeling: In duration judgments, for instance, the central-tendency bias is well predicted by optimal integration of the sample distribution and the sensory input (Jazayeri & Shadlen, 2010; Raviv, Ahissar, & Loewenstein, 2012; Shi & Burr, 2016; Shi, Church, & Meck, 2013).

While influences of ensemble statistics on time perception have been demonstrated mainly in studies of duration reproduction or temporal averaging (Acerbi, Wolpert, & Vijayakumar, 2012; Burr, Della Rocca, & Morrone, 2013; Chen et al., 2018; Ren et al., 2020; Zimmermann & Cicchini, 2020), duration context plays a critical role in duration comparison (Fründ, Wichmann, & Macke, 2014; Rhodes & Di Luca, 2016; Zimmermann & Cicchini, 2020), such as in the temporal-bisection task. In a typical temporal-bisection task, participants are given one *short* and one *long* duration as standards and they are asked to judge whether a given duration is closer to the *short* or the *long* standard (Allan & Gibbon, 1991; Raslear, 1985). Initially, researchers thought only the short and long standards matter in the temporal-bisection task, given that the task is to compare a sample duration to the both standards. However, it turned out that the sample durations themselves matter significantly (Brown, McCormack, Smith, & Stewart, 2005; Penney, Brown, & Wong, 2014; Wearden & Ferrara, 1995). For example, Wearden and Ferrara (1995) found that with the same short and long standards, a logarithmically spaced duration set, as compared with a linearly spaced set, had a lower *bisection point* (the time point that is subjectively equally distant to the short and long standard)—an effect that has been referred to as “spacing effect.” The main account for the spacing effect holds that the temporal-bisection task does not really involve comparing a sample duration to the short and long standards, but rather to a reference point *M* somewhere near the geometric or the arithmetic mean of the two standards (Wearden & Ferrara, 1995). Brown et al. (2005) subsequently found that using one reference point is not sufficient to explain the contextual bias. Rather, other factors, in particular the rank of the duration in the sampled set, do also matter—that is, the same duration in two different sets (with the same short and long standards) led to different bisection points when the order (percentile) of the duration in the two sets was different. Drawing on range frequency theory (RFT; Parducci, 1963) to combine the two factors of the relative range and the order of the sampled durations, Brown et al. (2005) proposed that the subjective judgment of a given time interval change is based on the weighted average of its range position (i.e., how far it is from the short and long standards) and its rank order in the distribution. Although temporal RFT (TRFT; Brown et al., 2005) successfully captures the biases in the temporal-bisection task, it still lacks a plausible theoretical explanation of how we actually process ranks and allocate weights to the relative range position and the relative rank. This approach would require observers to store individual durations and their relative orders in the set, which becomes extremely difficult, if not impossible, when the set size increases, and even worse when the same duration may be perceived differently across trials.

One possible alternative, and straightforward, account might be that instead of storing individual durations to calculate their orders, observers use ensemble statistics from a longer-term memory of the sampled durations (Cicchini, Arrighi, Cecchetti, Giusti, & Burr, 2012) to estimate the *M*-reference (i.e., bisection point) in the bisection task. Remembering individual items is difficult, whereas representing ensemble statistics is quick and intuitive (Alvarez, 2011; Ariely, 2001). Use of ensemble representations has been shown for various types of features, such as the average speed of moving objects (Watamaniuk & Duchon, 1992), the average size of objects (Marchant, Simons, & de Fockert, 2013), and the average emotional (facial) expression of a crowd of people (Haberman & Whitney, 2009). Given the limited capacity of attention and memory, we have to consciously identify and remember the plethora of objects and events we continually encounter, ensemble representations provide us with a ready means to bolster our perceptual experience (for reviews, see Cohen, Dennett, & Kanwisher, 2016; Whitney & Yamanashi Leib, 2018). In the temporal domain, it has been shown that people can learn ensemble statistics of time intervals up to the third central moment (i.e., mean, variance, and skewness) and use these statistics in their decision-making (Acerbi et al., 2012). Thus, in the temporal-bisection task, observers likely compare a perceived duration to the ensemble representation of the sampled distribution, rather than storing (and adjusting) individual ranks for later comparison. That is, acquiring the ensemble statistics of the test intervals, observers make bisection judgments according to the location of a given test interval within the learnt distribution. We refer to this as the ensemble-distribution account (EDA)*.*

One strong difference of EDA to previous proposals (e.g., the spacing account) is that EDA takes the shape of the distribution into account in making bisection decisions. Accordingly, EDA would predict a shift of the bisection point when the shape of the distribution changes while the spacing of the probe durations remains the same. In addition, EDA would predict the variance of the ensemble statistics to influence the difficulty of temporal judgment (measured by the slope of the psychometric curve). On these grounds, we conducted three experiments to test the predictions of the ensemble-distribution account. Specifically, Experiment 1 was designed to examine for the shift of the bisection point in sets with positively skewed (PS) versus negatively skewed (NS) spacing. In this regard, EDA makes the same prediction as, and so would be indistinguishable from, the spacing and TRFT accounts. Experiment 2 further examined the bisection task with equally spaced durations under two skewed frequency-distribution sets (see Fig. 1 for details): ascending frequency (AF) and descending frequency (DF). Given that the two sets have different ensemble means, we expected EDA to be able to predict, and account for, the difference in bisection points between the two conditions. Finally, Experiment 3 manipulated the variability of the sample distributions while keeping the mean of the distributions the same, by introducing a U-shaped and an inverted T-shaped set, with the former having a greater variance than the latter. According to EDA, the variance would influence the difficulty of the bisection (reflected in the JND), rather than the bisection point (reflected in the PSE). Additionally, we applied hierarchical Bayesian modeling to the behavioral data according to various assumptions of how temporal bisection may be performed. The aim of the model fitting and comparison was to look at the data patterns obtained in the three experiments with respect to the manipulations of stimulus spacing, distribution means, and variances, so as to identify the best possible account of how the ensemble context modulates performance of the bisection task.

**Fig. 1:**
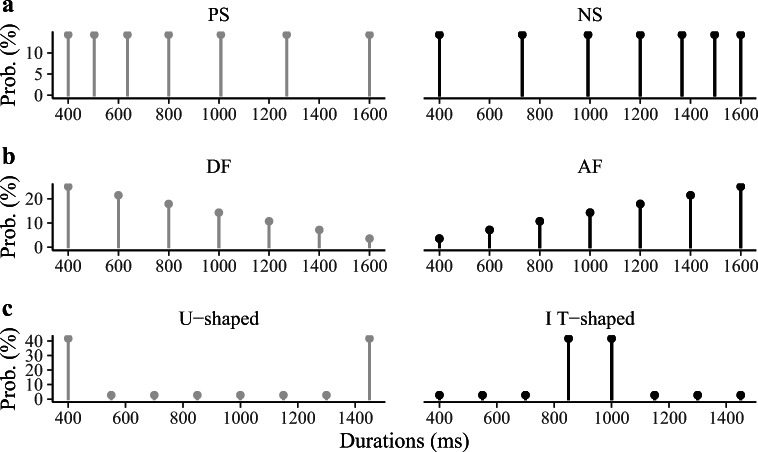
Sample distributions used in Experiments 1, 2, and 3. **a** Two spacing conditions used in Experiment 1: In the positively skewed session (PS), the intervals are spaced logarithmically between 400 and 1,600 ms, with a mean of 888 ms and a standard deviation (*SD*) of 401 ms; in the negatively skewed session (NS), the intervals follow a mirrored logarithmic spacing, with a mean of 1,112 ms and an *SD* of 401 ms. **b** Two sample-frequency conditions for the seven equally spaced intervals (400, 600, 800, 1000, 1200, 1400, 1600 ms) used in Experiment 2: The descending frequency (DF) session has an (arithmetic) mean of 800 ms and an *SD* of 347 ms; the ascending frequency (AF) session has the mean of 1,200 ms and an *SD* of 347 ms. **c** Two types of sample-frequency conditions for the eight equally spaced intervals (400, 550, 700, 850, 1000, 1150, 1300, 1450 ms) used in Experiment 3: The U-shaped session has a mean of 925 ms and an *SD* of 491 ms; the inverted T-shaped session has a mean of 925 ms and an *SD* of 175 ms

## Methods

### Participants

Forty-five university students with normal hearing took part in three experiments (15 each in Experiments 1, 2, and 3; 25 females; mean age: 25.5 years). The sample size was determined based on the prior study of Penney et al. (2014). Although they did not report effect sizes, we calculated *η*_*g*_= 0.27 based on their report of a one-way analysis of variance (ANOVA) test for the auditory condition. With α = .05, 1 − β = .85, and a within-subject (repeated-measures) ANOVA design, the sample size required for replicating this effect is nine observers. Taking a conservative approach, we opted for a sample size of 15 participants. All participants gave written consent according to the institutional guidelines prior to their participation and were paid 9 euro per hour for their service. The study protocol was approved by the LMU Faculty of Pedagogics & Psychology Ethics Board. All participants were naïve as to the purpose of the study.

### Stimuli and apparatus

The experiments were conducted in a sound-reduced and moderately lit test room. Stimuli were generated by Psychtoolbox-3 (Kleiner, Brainard, & Pelli, 2007) based on MATLAB R2014a (The MathWorks, Inc., Natick, MA). Auditory stimuli were generated by PsychPortAudio on a HP ProDesk computer and presented through the loudspeakers. Participants gave their responses by pressing the left or right arrow keys on the keyboard. Experimental instruction and feedback information were presented on a CRT monitor.

### Procedure

We adopted the bisection task in all three experiments. Participants were familiarized with the task in two practice blocks prior to the main experiment (56 trials per block in Experiments 1 and 2; 72 trials per block in Experiment 3). The practice blocks involved the same procedure as the experiments proper, except that (i) all test intervals were uniformly distributed (i.e., equally frequent), whereas the two distributions compared and contrasted in the formal Experiments 2 and 3 were nonuniform; and (ii) response feedback, referenced to the average of the short and long standards (see next paragraph), was provided on each trial, whereas no feedback was given in the formal experiments. Note that in Experiment 1, the intervals presented during practice had the skew inherited from the duration spacing in the formal experiment (see subsection Experiment 1, below, for details), but the feedback reference was the same for all practice trials. Thus, if there were any effects of the training conditions in the practice blocks—in particular, assimilation of the PSE to the (common) feedback reference or sharpening of the psychometric curve—they would be the same for the two conditions that we manipulated in the experiment proper and so work against finding the predicted differential effects between the two conditions in the formal experiment. Thus, any differential effects we observe are unlikely confounded by practice effects.

A trial started with a visual fixation marker and a brief beep (20 ms, 1000 Hz, 60 dB), followed by a blank display of 500 ms, prompting participants to get ready for a new trial. Next, a white-noise stimulus (60 dB) was presented for a given duration, randomly selected from a predetermined set, ranging from 400 to 1,600 ms (seven test intervals in Experiments 1 and 2, eight intervals in Experiment 3; see next paragraph for details). Immediately following the offset of the white-noise stimulus, a display with a question mark (“?”) was shown, prompting participants to indicate whether the duration of the stimulus was closer to the *short* standard or the *long* standard by pressing the left or the right arrow key, respectively. In the practice block, participants received feedback after their responses (i.e., either “The presented interval was close to the short standard” or, respectively, “The presented interval was close to the long standard,” depending on whether the interval was shorter or longer than the average of the short and long standards)*.* When the test interval coincided with the mean of the short and long standards (1,000 ms in Experiment 2), a random feedback (50% “The presented interval was close to the short standard” and 50% “The presented interval was close to the long standard”) was given. In the formal experiment, participants received no feedback regarding their responses. After a blank interval of 900 to 1,100 ms, the next trial began.

#### Experiment 1

For better comparison, the sets of intervals introduced in Experiment 1 were similar to the set used in the (Penney et al., 2014) study, in which logarithmic spacing of durations between the short and long standards was applied. As depicted in Fig. 1a, the seven durations used in the positively skewed (PS) session were 400, 504, 636, 800, 1008, 1270, and 1600 ms, and in the negative skewed (NS) session 400, 730, 992, 1200, 1366, 1496, and 1600 ms. Each duration was repeated 48 times in the session (i.e., the durations were distributed uniformly), and the seven durations were presented randomly intermixed within each session. Each participant completed two sessions, each consisting of six blocks of 56 trials. The order of sessions was counterbalanced (as well as possible) across participants.

#### Experiment 2

Two types of sample-frequency distributions were tested in separate sessions: a descending sample frequency (DF) and an ascending sample frequency (AF), as depicted in Fig. 1b. In the AF session, the sample frequencies were (1/28, 2/28, 3/28, 4/28, 5/28, 6/28, 7/28) for the intervals (400, 600, 800, 1000, 1200, 1400, 1600 ms); the DF session, the sample frequencies were reversed. There were six blocks of 56 trials in each session.

#### Experiment 3

Eight intervals between 400 and 1,450 ms (400, 550, 700, 850, 1000, 1150, 1300, and 1450 ms) were used in Experiment 3. Two types of sample frequencies were implemented: A U-shaped distribution and an inverted T-shaped distribution (see Fig. 1c). In the U-shaped sampling session, the presentation frequencies of durations (400, 550, 700, 850, 1000, 1150, 1300, and 1450 ms) were (30/72, 2/72, 2/72, 2/72, 2/72, 2/72, 2/72, 30/72), respectively; in the inverted T-shaped sampling session, the frequencies were (2/72, 2/72, 2/72, 30/72, 30/72, 2/72, 2/72, 2/72) for the same durations. The two types of sample distributions have the same arithmetic mean, but they differ in their variability. There were four blocks of 72 trials in each session. The order of the test intervals was randomized within each session, and the order of the sessions was counterbalanced (as well as possible) across participants.

### Statistical analysis

R package quickpsy (Linares & López-Moliner, 2016) was used to fit psychometric functions and calculate the points of subjective equality (PSE, here the bisection point) and the just noticeable differences (JNDs). We used the cumulative Gaussian function as the psychometric function and the standard deviation of the estimated function as the JND (i.e., the difference between the thresholds at 50% and 75%). All statistical tests were conducted using repeated-measures ANOVAs—with additional Bayes-factor analyses to comply with the more stringent criteria required for acceptance of the null hypothesis (Kass & Raftery, 1995; Rouder, Speckman, Sun, Morey, & Iverson, 2009).

## Results

### Spacing effect in distributions with different ensemble means (Experiment 1)

**Fig. 2:**
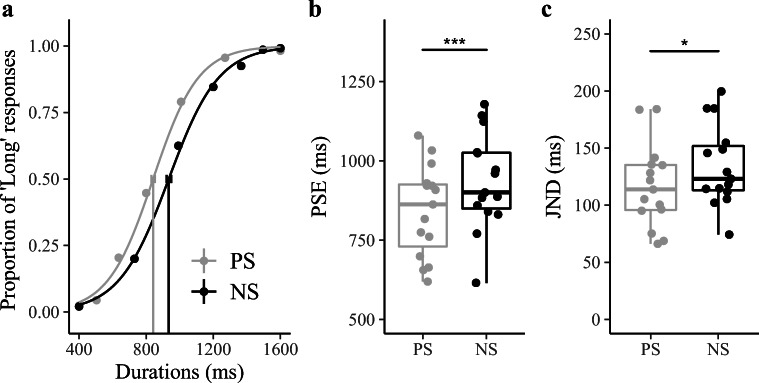
Results of Experiment 1. **a** Bisection functions (proportions of “long” responses plotted against the comparison durations, and fitted psychometric curves) averaged across 15 participants for the two, positively (PS) and negatively skewed (NS), stimulus-spacing conditions. **b** Boxplots of PSE of the duration judgments for the PS and NS sessions (***p < .001). The dots depict individual PSEs estimated from individual participants. Lower and upper tips of the vertical lines correspond to minimum and maximum values, the box represents the interquartile range (between 25% and 75%), and the horizontal line represents the median. **c** Boxplots of JND of the duration judgments for the PS and NS sessions (*p < .05). The dots depict individual JNDs of individual participants

The psychometric functions, depicting the relation between the proportion of “long” responses and the test durations, are illustrated in Fig 2a. By visual inspection, participants made more “long” responses in the PS session. This was confirmed by an analysis of the PSEs: As depicted in Fig 2b, the mean PSEs (±standard error, *SE*) for the PS and NS sessions were 842 (±38) ms and 934 (±40) ms, respectively. A repeated-measures ANOVA revealed this effect of the spacing condition to be significant, *F*(1, 14) = 21.141, *p < .*001, *η*_*g*_= .0943, *BF* = 47.4. Thus, Experiment 1, which used the similar stimulus settings and procedure as Penney et al. (2014), replicated their spacing effect, as expected (Brown et al., 2005; Penney et al., 2014).

As shown in Fig. 2c, the mean JNDs (±*SE*) were 116.8 (±9.7) ms for the PS and 134.1 (±9.4) ms for the NS distribution. A repeated-measures ANOVA revealed the difference to be significant, *F*(1, 14) = 5.755, *p* = .031, *η*_*g*_ = 0.059, *BF* = 2.10. The larger JND in the NS versus the PS condition is likely attributable to Weber scaling in the perceived durations. Subjective time is known to roughly follow Weber’s law (i.e., it exhibits the scalar property), with longer durations showing larger variability of the subjective estimates than shorter durations (Gibbon, 1977; Wearden & Lejeune, 2008). As there were more longer durations in the NS session than in the PS session (see Figure 1a), the uncertainty in the NS session was likely higher, giving rise to the increased JNDs relative to the PS session.

### Shifts in BPs are associated with ensemble mean but not with spacing information (Experiment 2)

**Fig. 3:**
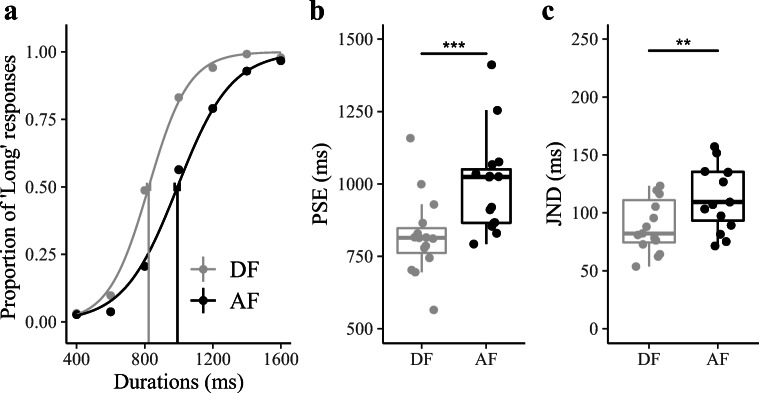
Results of Experiment 2. **a** Bisection functions (proportions of “long” responses plotted against the comparison durations, and fitted psychometric curves) averaged across 15 participants for the two, descending-frequency (DF) and ascending-frequency (AF), duration-distributions conditions. **b** Boxplots of PSE of the duration judgments for the DF and AF conditions (***p < .001). The dots depict individual PSEs estimated from individual participants. **c** Boxplot of JND of the duration judgments for DF and AF conditions (**p < .01). The dots depict individual JNDs of individual participants

In Experiment 2, distributions of descending frequency (DF) and ascending frequency (AF) were generated for the same set of durations with equal spacing (step of 200 ms). The arithmetic mean was 800 ms in the DF condition, versus 1200 ms in the AF condition. Figure 3a depicts the average psychometric curves for the two (DF and AF) conditions, showing a marked shift in the location of the bisection points: the mean PSE (±SE) was 997±45 ms for the AF condition and 821±37 ms for the DF condition (see Fig. 3b). This difference was significant, repeated-measures ANOVA on the PSEs: *F*(1, 14) = 26.83 *p* < .001, *η*_*g*_ = .263, *BF* = 222.0. That is, compared with the AF condition, the DF condition was associated with an increased probability of “long” responses, the latter consisting of relatively more *short* intervals and thus having a relatively shorter arithmetic mean of the stimulus set (which serves as a reference for the bisection). According to EDA, temporal bisection essentially involves a comparison of a given duration to the estimate of the ensemble mean. Thus, compared with the AF set, the relatively *shorter* ensemble mean in the DF set would lead to more “long” responses.

As shown in Fig. 3c, the mean JNDs were 108 (±22.7) ms for the DF condition and 138.8 (±28) ms for the AF condition, with the difference being significant (repeated-measures ANOVA): *F*(1, 14) = 15.38, *p* < .01, *η*_*g*_ = .027, *BF* = 17.82. Similar to Experiment 1, the frequency distribution with more *long* durations (here, the AF session) had a larger JND than the distribution with more *short* durations. Again, this was likely due to unequal Weber scaling in the two sets: the uncertainty induced by the *long* durations was more prominent in the AF (as compared with the DF) session, further pointing to the influence of ensemble statistics in duration comparison.

### Sensitivity of temporal judgment is driven by ensemble variance (Experiment 3)

**Fig. 4:**
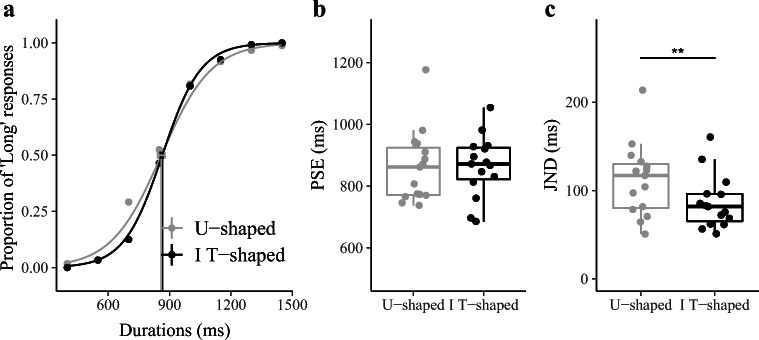
Results of Experiment 3. **a** Bisection functions (proportions of “long” responses plotted against the comparison durations, and fitted psychometric curves) averaged across 15 participants for the two, U-shaped and inverted T-shaped, frequency-distribution conditions. **b** Boxplots of PSE of the duration judgements for the U-shaped and inverted T-shaped frequency distributions. **c** Boxplots of JND of the duration judgments for the U-shaped and inverted T-shaped frequency distributions (**p < .01)

Experiment 3 was designed to examine whether performance on the temporal-bisection task would be affected by the variance of the contextual stimulus set. Accordingly, the distributions of the stimulus set (both with equal spacing of the interval durations) had the same mean, but they differed in their variance. Figure 4a depicts the psychometric functions averaged across 15 participants for each stimulus set condition: U-shaped and inverted T-shaped frequency distribution. Consistent with the prediction of EDA, the two psychometric curves cross each other at the 50% threshold, while having different slopes. As depicted in Figure 4b, the mean PSEs (±*SE*) were comparable: 863 (±31.5) ms and 864 (±26.5) ms for the U-shaped and inverted T-shaped distributions, respectively (repeated-measures ANOVA): *F*(1, 14) = 0.001, *p* = .97, *η*_*g*_= .000026, BF = 0.331, with the BF value providing strong evidence in favor of the null hypothesis). The absence of a difference in the PSEs between the U-shaped and inverted T-shaped distributions in this experiment, combined with findings from Experiments 1 and 2, suggests that the shift in PSEs was driven mainly by the ensemble mean, not the ensemble variance.

In contrast, as can be seen from Fig. 4c, there was a marked difference in the JNDs between the two conditions, with mean JNDs of 111.75 (±11) ms for the U-shaped and 86.37 (±8) ms for the inverted T-shaped distribution. A repeated-measures ANOVA revealed the difference to be significant: *F*(1, 14) = 9.171, *p* < .01, *η*_*g*_= .117, *BF* = 5.96.

Following the prediction of EDA, we expected a significantly steeper slope (i.e., a smaller JND) with the inverted T-shaped, as compared with the U-shaped, frequency distribution, given that the variance was smaller in the former than in the latter. In addition to the variance of the set, the Weber scaling of the longest duration may also contribute to the large variance in the U-shape condition, similar to the differences in JNDs that we observed in Experiments 1 and 2.

## Modeling

**Fig. 5:**
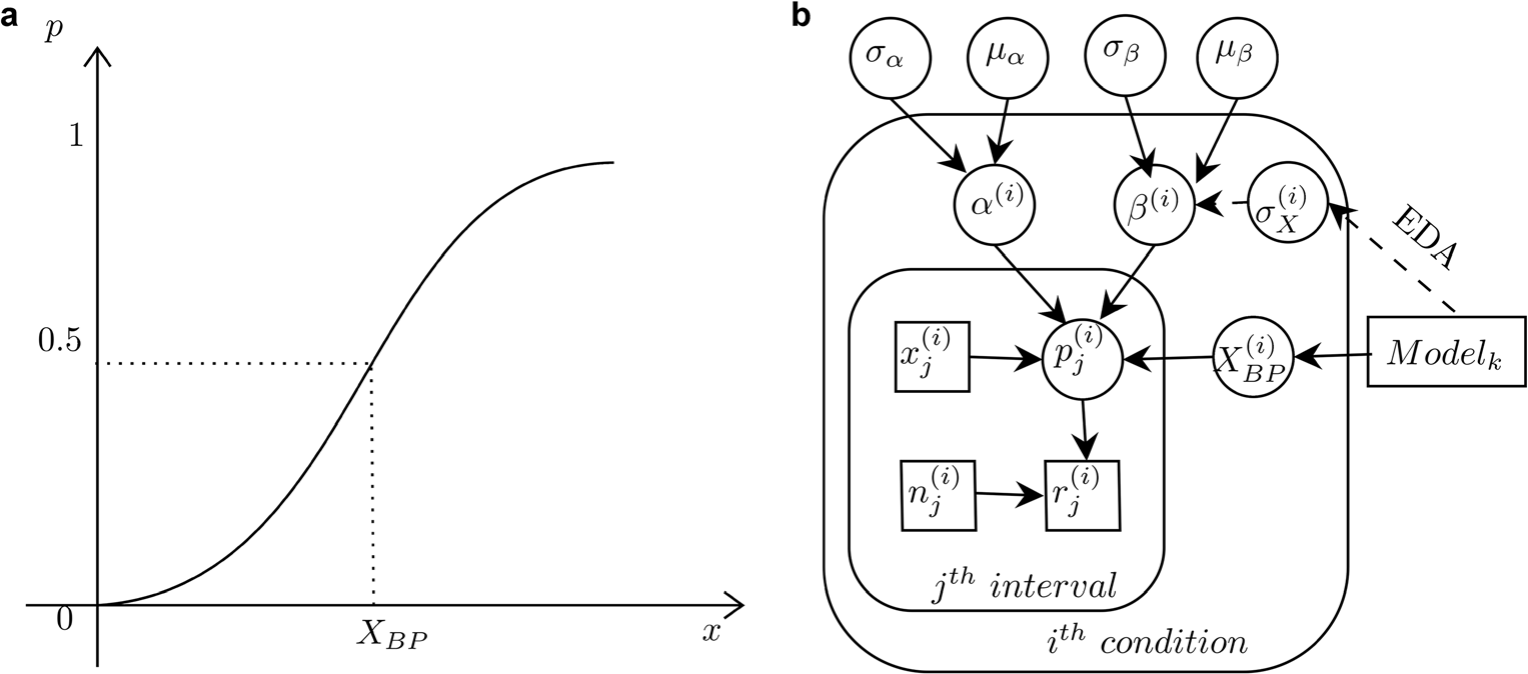
The framework of the hierarchical model. **a** A psychometric function with bisection point *X*_*BP*_. **b** Schematic illustration of the hierarchical model of temporal duration judgments (see text for details)

To compare different models of the temporal-bisection task, we applied Bayesian hierarchical modeling (Lee & Wagenmakers, 2014) to our behavioral data, with correspondent assumptions about how the task is performed. The framework of the hierarchical model is illustrated in Fig. 5. For a given duration ${X}_j^{(i)}$ in condition *i*, we assume the bisection response follows the binomial distribution for the probability of the “long” response ${p}_j^{(i)}$. The probability ${p}_j^{(i)}$ is determined by the ratio comparison of probe duration ${X}_j^{(i)}$ and the bisection point ${X}_{BP}^{(i)}$ according to the following psychometric relation:

1 $$ \mathit{\log}\frac{p_j^{(i)}}{1-{p}_j^{(i)}}={\alpha}^{(i)}+{\beta}^{(i)}\left({X}_j^{(i)}/{X}_{BP}^{(i)}-1\right), $$

where *α*^(*i*)^ and *β*^(*i*)^are two psychometric parameters. The left side of the equation is the decision variable expressed in the log-likelihood of the two alternative (“Long” vs. “Short”) responses, while the right side assumes the comparison is based on the ratio *X*_*j*_/*X*_*BP*_. The ratio comparison on a linear scale is equivalent to the subtraction comparison on the internal log-scaled representation, which conforms to Weber scaling (i.e., the scalar property). The assumption of a logarithmic scale for internal duration representation is common in theories and models of duration judgments (Petzschner, Glasauer, & Stephan, 2015; Ren, Müller, & Shi, 2020; Roach, McGraw, Whitaker, & Heron, 2017; Wearden, 1991). The equation implies that the decision variable is a linear function of the ratio comparison. The coefficient *β*^(*i*)^ reflects both the sensitivity of the ratio comparison and a potential central-tendency bias (which makes the slope of the psychometric function shallower) for individual participants, in a given condition *i*. In Bayesian hierarchical models, the parameters *α*^(*i*)^ and *β*^(*i*)^ are assumed to follow Gaussian distributions with the respective hyperparameters (*μ*_*α*_, *σ*_*α*_) and (*μ*_*β*_, *σ*_*β*_). In addition, EDA assumes that the distribution of decision sensitivity *β*^(*i*)^ is Gaussian with a mean proportional to the reciprocal of the relative spread of the test durations, that is: *N*(*k* · *μ*/*σ*_*X*_, *σ*_*β*_), where *μ* and *σ*_*X *_are the mean and, respectively, the standard deviation of the test durations, and *k* is a scaling factor. In other words, narrower sample distributions would enhance the sensitivity of the bisection task. It should be noted that, as implemented, our EDA model uses the veridical spread of the sampled duration *σ*_*X*_, disregarding the subjective Weber scaling in the perceived ensemble variability ${\sigma}_X^{\prime }$; but the framework can be easily extended to the subjective scale.

The main difference among the various models, ranging from the simple bisection model to EDA, is how the critical reference ${X}_{BP}^{(i)}$ (reference *M* in Wearden & Ferrara, 1995) is used in the bisection task (see Table 1). The simple bisection model assumes the comparison is made either between the ratios *X*_*S*_/*X* and *X*/*X*_*L*_ or between probe *X* and the arithmetic-mean duration (*X*_*L*_ + *X*_*S*_)/2; that is, essentially the comparison is made to either the geometric mean (GM) or the arithmetic mean (AM) of the standard durations. From their meta-analysis of 148 experiments, Kopec and Brody (2010) concluded that it remains controversial whether the bisection point is close to the GM or AM. The bisection point is influenced by a number of factors, including the short-long spread (i.e., the Long/Short ratio) and the probe context. The spacing account, for example, assumes the comparison reference is the arithmetic mean of the whole probe durations, each of which is equally frequent by design (Penney & Cheng, 2018). The TRFT account (Brown et al., 2005; Penney et al., 2014), on the other hand, assumes that, rather than being veridical, subjective duration is an average of two components—namely, the relative position of a sampled duration in-between the short and long standards and the ordinal position of the duration within the sample durations. The proportion of “long” responses to a sampled duration is then based on a comparison between its calculated relative position and the short and long standards. Accordingly, the estimated temporal bisection point ${X}_{BP}^{(i)}$ lies roughly between the mean of the sample distribution and the arithmetic mean of the short and long standards. We simulate this using the ensemble-mean model, that is: ${X}_{BP}^{(i)}$ is the mean (either the GM or the AM) of the sampled distribution. Given that the mean of the sample distribution is not a fixed parameter known to participants, but rather updated dynamically over the course of the trials, we further propose that the bisection reference (${X}_{BP}^{(i)}$) is also a variable that fluctuates from trial to trial, while being centered around the geometric or the arithmetic mean of the sampled distribution. We refer to this as the two-stage ensemble-mean model (see Table 1). The EDA model goes one step further, by incorporating the variance of the distribution into bisection decisions—that is, the slope of the psychometric function (*β*) is inversely related to the relative spread of the ensemble distribution (*σ*_*X*_/*μ*; see Table 1). EDA predicts that increasing the variability of the test durations would decrease the bisection sensitivity when the mean of the test range is fixed. Moreover, given that EDA (see Equation 1) conforms to Weber scaling (Jozefowiez, Polack, Machado, & Miller, 2014; Kopec & Brody, 2010; Wearden & Lejeune, 2008), *β* would remain unchanged when the ratio of the mean and standard deviation of the test durations is kept constant.

**Table 1:** Models and model assumptions about the comparison reference *X*_*BP*_ and decision sensitivity (slope)

Models	Reference *X*_*BP*_	Decision sensitivity *β*
Simple bisection model	AM: *X*_*BP*_ = (*X*_*s*_ + *X*_*L*_)/2 GM: ${X}_{BP}=\sqrt{X_s{X}_L}$	N/A
Spacing model	AM: ${X}_{BP}=\frac{1}{n}\sum {X}_j$	N/A
	GM: ${X}_{BP}=\sqrt[n]{\varPi {X}_j}$	
Ensemble-mean model	AM: ${X}_{BP}=\frac{1}{\varSigma {f}_j}\sum {f}_j{X}_j$	constant^*^
	GM: ${X}_{BP}=\sqrt[\varSigma {f}_j\ ]{\varPi\ {\left({X}_j\right)}^{f_j}}$	
Two-stage ensemble-mean model	AM: *X*_*BP*_ ∼ *N*(*μ*, *σ*), $\mu =\frac{1}{\varSigma {f}_j}\sum {f}_j{X}_j$	constant
	GM: *X*_*BP*_ ∼ *N*(*μ*, *σ*), $\mu =\sqrt[\varSigma {f}_j\ ]{\varPi\ {\left({X}_j\right)}^{f_j}}$	
Ensemble-distribution account (EDA)	AM: *X*_*BP*_ ∼ *N*(*μ*, *σ*), $\mu =\frac{1}{\varSigma {f}_j}\sum {f}_j{X}_j$	*β* ∼ *N*(*k* · *μ*/*σ*_*X*_, *σ*_*β*_)
	GM: *X*_*BP*_ ∼ *N*(*μ*, *σ*), $\mu =\sqrt[\varSigma {f}_j\ ]{\varPi\ {\left({X}_j\right)}^{f_j}}$	

*Note*. * TRFT model (Brown et al., 2005; Penney et al., 2014) used the percentile to approximate the subjective magnitude, and then applied ratio comparison between the perceived magnitude to the short and long standards, which implicitly incorporates some degree of variance of the distribution in the bisection decision. N/A denotes not applicable

**Fig. 6:**
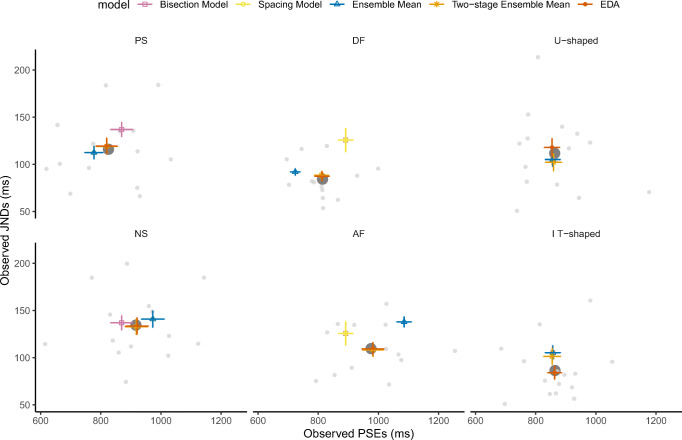
Observed PSEs and JNDs and mean predictions from the models compared. PSE–JND pairs from individuals are plotted in gray dots, and their means are shown in large dark dots. The mean predictions of PSE–JND pairs and their PSEs from the five models are marked with colored error bars. Note that the predictions of the spacing model and the ensemble-mean model were the same (overlapped in the figure) for the PS and NS sets (the left panel), given that the sampled durations were equally weighted. Due to the equal spacing in the DF, AF, U-shaped, and IT-shaped sets, the bisection and the spacing models made the same predictions (overlapped in the figure). The predictions from the EDA model came the closest to the mean of the observed PSE–JND pairs across all six sets

Based on those assumptions, we fitted the above five models to our data and estimated the corresponding psychometric functions, PSEs, and JNDs for each participant. We applied the AM and GM as ${X}_{BP}^{(i)}$ in all models. The simple bisection model and the spacing model lowered the PSE with the GM relative to the AM, while both the two-stage ensemble-mean model and the EDA model yielded very close predictions with both the GM and the AM (the relative mean difference between the two predictions of the PSEs $\frac{\left| PS{E}_{GM}- PS{E}_{AM}\right|}{PS{E}_{obs}}$ was less than 2%), owing to the trial-by-trial variation rendering the small difference between the GM and AM of little effect in the hierarchical model. Overall, EDA outperformed all other models (see below), whether incorporating the AM or GM variant. Given this, here, we consider only the models with the AM. To visually compare the various models with regard to their respective predictions of the PSEs and JNDs, we plotted their mean predictions with individual estimates in Fig. 6. The spacing model and the ensemble-mean model made the same prediction for the spacing manipulation in Experiment 1 (PS vs. NS), given that the sampled durations were weighted equally (shown overlapped in the left panel of Fig. 6). Both, however, underestimated the PSE and JND in the PS condition (more short durations), and overestimated the PSE and JND in the NS condition (more long durations). This suggests that the bisection point (BP) was assimilated to the mean of the skewed distribution, though only partially. By contrast, the two-stage ensemble-mean model and the EDA model provided a very close prediction to the observed PSEs in Experiment 1. In addition, the EDA model (but not the two-stage ensemble-mean model) predicted the mean JNDs. The main difference between the two-stage ensemble-mean (and EDA) model(s) and the ensemble-mean model is that the former assumes random trial-to-trial fluctuations of the ensemble mean, thus potentially incorporating a partial range effect in the decision (i.e., assimilation to the arithmetic mean). The ensemble means were more extreme in the DF and AF conditions in Experiment 2 as compared with those in Experiment 1, deviating greatly from the observed PSEs (see the middle panel in Fig. 6). This suggests that the mean of the distribution was not the sole factor determining the bisection judgments. Again, by having the reference (i.e., bisection point) vary across trials, both the two-stage ensemble-mean model and the EDA model well predict the observed PSEs. Incorporating the variance of the distribution in the bisection decision, the EDA model was able to predict the JNDs across all six different sets, indicating that the second moment of the ensemble statistics (i.e., variance) does influence performance of the bisection task.

**Fig. 7:**
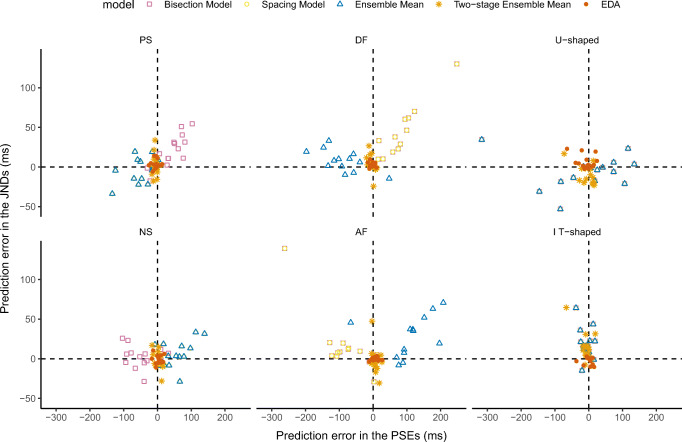
Scatterplot of prediction errors in the PSEs and JNDs derived from the five models for individual observers across the six duration sets presented in Experiments 1–3 (see Fig. 1). Perfect predictions are located at the center (0, 0). The average prediction errors of the six conditions of the EDA model were the smallest

To obtain a better picture of the model predictions at the level of individual observers, we plotted the prediction errors (predicted vs. observed values) in PSEs versus JNDs in Fig. 7. Each point represents the errors in PSE and JND estimation, per individual participant, derived from a specific model of the five models compared. As can be seen from Fig. 7, the scatter points of the EDA model are centered nearer to the origin of the *XY* coordinates compared with the points of the other models, indicating that the PSEs and JNDs predicted by EDA come closest to the mean of the observed values, across all three experiments. This observation is supported by measures of the Euclidean distances of the model predictions from the observed PSE-JND pairs: The mean distances were 68.4, 68.1, 75.2, 16.7, 11.9 ms for the simple bisection, spacing, ensemble mean, two-stage, and EDA models, respectively. Formal corroboration of the superiority of EDA is provided by “goodness-of-fit” measures of the predicted psychometric curves using the Watanabe–Akaike information criterion (WAIC). The WAIC is a measure of the quality of a hierarchical model, which takes into account the goodness of fit, as measured by the likelihood, while also penalizing models with more free parameters (Vehtari, Gelman, & Gabry, 2017). Lower WAIC values indicate better model performance. The mean WAICs for the predicted “long” responses across all test durations were 77.2, 92.7, 104.6, 76.7, and 72.1 for the simple bisection, spacing, ensemble mean, two-stage, and EDA models, respectively. That is, across all conditions, the EDA model provides the best fit for both the PSEs and the JNDs, evidenced by the fact that the WAIC for the EDA model was the smallest in all cases.

## General discussion

In the present study, we tested six different duration sets, with set properties varying in stimulus spacing, set mean, and set variance in three experiments, to investigate whether temporal-bisection judgments would be best explained by our ensemble-distribution account (EDA). Experiment 1 demonstrated that the skewness of unequal spacing significantly shifted the bisection point, confirming previous findings (Brown et al., 2005; Penney et al., 2014; Wearden & Ferrara, 1995). Given that the short and long standards were identical in the two (positively skewed (PS) and negatively skewed (NS)) sets, the finding of differential PSEs between the PS and NS conditions argues against the simple bisection account, irrespective of whether it assumes the arithmetic or the geometric mean of the standards as reference. Experiment 2 further demonstrated that the frequencies of the sampled durations greatly impacted the bisection point, even when the probe durations were equally spaced in the ascending-frequency (AF) and descending-frequency (DF) sets. The spacing account fails to predict this effect. Experiment 3 kept the spacing of the durations and the mean of the sets the same for the U-shaped and inverted T-shaped sets, but varied their variances (larger variance for the U-shaped set). The results revealed the variance of the set to influence the sensitivity of temporal bisection, reflected in differential JNDs (despite the equivalent set means). While previous accounts failed to predict the PSEs and JNDs in one set or another, the EDA model successfully accounted for the shifts of the PSEs and JNDs in all six sets examined. The results of model-fitting analyses also showed the EDA model to provide the best account of the data.

### Temporal bisection and related accounts

The temporal-bisection task was first developed in research on animal timing (Gibbon, 1977; Gibbon et al., 1984) and later adapted to studies on human timing (Allan & Gibbon, 1991; Wearden, 1991). The focus of the initial studies on temporal bisection was on how humans and other animals make interval comparisons (rather than on context-dependent manipulations of the bisection point). Accordingly, the early work implicitly assumed that the interval comparison in temporal bisection depends only on the probe stimulus (*t*) and the short (*S*) and long (*L*) standards (e.g., Allan & Gibbon, 1991). Later studies, however, revealed that temporal bisection is sensitive to the probe context. Logarithmic versus linear spacing of the probe durations often resulted in different bisection points, even though the short and long standards remained the same (Brown et al., 2005; Penney et al., 2014). This raised the question as to the temporal reference to which observers actually compared a given probe duration. Using various spacing manipulations to probe for shifts of bisection points, Wearden and Ferrara (1995) suggested that observers likely compare the probe duration *t* to a reference *M*, rather than with *S* and *L*, where *M* lies somewhere in-between the geometric and arithmetic mean of the *S* and *L*. While this *M*-reference proposal could qualitatively explain the shifts of the bisection points, it falls short of quantitatively predicting the influence of the sampled distribution. Subsequently, Brown et al. (2005) developed the TRFT model based on the ‘range frequency theory’, arguing that the subjective measure of a given probe duration (*t*^′^) is influenced by its temporal position within the sample range and its percentile in the distribution. The TRFT model still considers temporal-bisection judgments to involve a ratio comparison between *S*/*t*^′^ and *t*^′^/*L*, where *S* and *L* are veridical. It should be noted that the TRFT model assumes that observers can retrieve the ordinal (rank) position of the probe duration (i.e., percentile), which logically requires a representation of the full ordered sequence of the durations. Building up such a representation would be very memory-intensive and thus quite unlikely with a large set of durations. By contrast, Wearden and Ferrara’s (1995) *M*-reference model only requires an estimation of *M* across all trials, which is computationally less intensive. Here, we adopted the *M*-reference approach to compare various models, assuming that temporal-bisection judgments are made by comparing the probe *t* to a bisection point *X*_*BP*_, with the *X*_*BP*_ being context sensitive. The model comparison revealed that the best fit is provided by the EDA account, which assumes that the bisection point is derived from the ensemble mean with trial-to-trial random variation. Given that the ensemble statistics are not known prior to the experiment, but rather updated dynamically from trial to trial, EDA, as well as the two-stage ensemble-mean model, can capture the dynamic adaptation of the bisection point, making them outperform the other models.

The assumption that the bisection point is sensitive to context does not rule out that subjective measures of individual probe durations themselves are modulated by the context. In fact, perceived durations are known to be subject to the central-tendency effect (Lejeune & Wearden, 2009; Shi et al., 2013). However, the central-tendency effect only assimilates individual durations toward the ensemble mean, which could degrade the sensitivity of the bisection task (given that the subjective distance to the mean is shortened), while leaving the bisection point unaffected. In other words, the bisection point is insensitive to the central-tendency effect. Given this and for the purpose of simplicity, we did not explicitly incorporate the central-tendency effect in our modeling (though it was an implicit factor influencing the general free parameter *β*; see the modeling section and Equation 1).

One strong prediction deriving from EDA, which sets it apart from the other accounts, is that the variability of the sampled distribution influences the sensitivity of bisection judgments, as corroborated empirically in Experiment 3. The duration set with the inverted T-shaped distribution had a lower variability compared with the U-shaped set, which led to the psychometric curve becoming steeper. The reason is that the ensemble representation of the inverted T-shaped distribution would be narrower than that of the U-shaped distribution, given that ensemble perception incorporates the external statistics. As a result, any probe durations that deviate from the ensemble mean would be more likely judged as short or long in the inverted T-shaped set than in the U-shaped set.

### Ensemble perception for temporal sequences

It should be noted that, while previous research on ensemble perception has primarily focused on summary statistics of the immediately, or simultaneously, available information, such as the average size of a set of objects (Alvarez, 2011; Whitney & Yamanashi Leib, 2018), the ensemble perception we refer to here concerns the statistical summary representation that is acquired through trial history. Real-world sensory inputs relevant to our behavioral goals do not always occur all at once, and we are often exposed to changing characteristics of goal-relevant objects. For instance, linguistic research has shown that both infants and adults make use of statistical computations for deciding which series of sounds establishes a word within a nonstop flow of spoken sounds (Newport & Aslin, 2000; Saffran, 2001). In particular, people are able to track the regularities in a series, or “group,” of sound elements, which render the predictiveness of one sound element onto another. For language acquisition and the learning of new languages, such probability-based computations provide important rhythmic temporal information (i.e., acoustic features such as duration and frequency of speech elements and summary statistics of sequences of speech sounds and silent intervals) for parsing the sequential sounds in speech. And more recently, Chen et al. (2018) showed that humans automatically derive the mean interval from a sequence of auditory beeps, which then cross-modally influences, or “patterns”, visual apparent motion. The present findings add to this evidence by demonstrating that temporal ensemble perception developed through trial history provides the “reference” for duration comparison.

One might ask why we need ensemble perception in the first place, when temporal tasks, such as bisection, can be accomplished with greater precision without the influence of ensemble statistics. To answer this question, we need to consider the fundamental roles of ensemble perception. The environment we live in does not comprise random objects and events, but rather has structure and regularity (Cohen et al., 2016). We are continually confronted with abundant information that is beyond our processing capacity. Accordingly, evolutionary pressures pushed us to utilize regularity—that is, ensemble-statistical—information to overcome the capacity limit (Ariely, 2001; Whitney & Yamanashi Leib, 2018). As a result, deriving such ensemble statistics became “intuitive” and automatic. In many situations, using intuitive ensemble perception can help us recover unattended events, spot outliers, or make predictions. For example, when listening to background music while working, one can rapidly spot a change in rhythm even if one’s focus is not on music. Yet, in some instances, implicitly using ensemble statistics would give rise to unintended biases, such as the PSE/JND shifts we report here. It should be noted that in the bisection task, observers are actually explicitly told to compare the probe duration to the short (*S*) and long (*L*) standard. The reason why participants use the ensemble distribution as the reference, rather than *S* and *L*, is likely owing to the fact that *S* and *L* contribute to the ensemble distribution in the same way as the other durations (Wearden & Ferrara, 1995).

### The role of variability in ensemble perception

Previous research on ensemble perception has largely focused on demonstrating humans’ ability to accurately estimate mean values from an array of objects or sensory features (Whitney & Yamanashi Leib, 2018). However, the variability of the sampled stimuli provides useful information about the range, stimulus spacing, and exceptional cases in the data set—hence, the variance statistic is a key component in ensemble perception. Although processing mean information eases the limitations of our perceptual experience by summarizing multiple items or features to an exemplar component (Alvarez, 2011), detecting similarities or, respectively, deviations among items is not solely based on the mean information, but may also benefit from complementary measures of stimulus range and variance (Haberman, Lee, & Whitney, 2015; Michael, de Gardelle, & Summerfield, 2014; Solomon, 2010). Thus, for example, ensemble variance has been suggested to be useful for identifying potential outliers or deviations in a crowd (Whitney & Yamanashi Leib, 2018). The results of the present study show that variance information is also exploited in temporal judgments: In temporal bisection, the variance of the sample distribution provides useful information for discerning the location of a probe duration relative to the ensemble mean, thus enhancing temporal sensitivity (as evidenced by the steepness of the psychometric curve).

## Conclusion

The ensemble context is an important determinant in time perception. The present paper reported three experiments in which we manipulated the distribution of auditory duration sets to determine factors that influence temporal-bisection performance. The results revealed the mean and variance of the stimulus set to be critical factors, producing shifts of the bisection point and, respectively, changes of the slope of the bisection curves. These findings demonstrate that the human timing mechanism involves an ensemble averaging process which works similarly to other perceptual properties in the visual and auditory domains. Moreover, we proposed an *ensemble-distribution accoun*t that explains in which way subjective judgments of time intervals vary according to the distribution summary statistics of the set mean and variance values.
